# Nonreciprocal Transverse Photonic Spin and Magnetization-Induced Electromagnetic Spin-Orbit Coupling

**DOI:** 10.1038/srep39972

**Published:** 2017-01-06

**Authors:** Miguel Levy, Dolendra Karki

**Affiliations:** 1 Physics Department, Michigan Technological University, Houghton, Michigan, USA; 2 Henes Center for Quantum Phenomena, Michigan Technological University.

## Abstract

We present a formulation of electromagnetic spin-orbit coupling in magneto-optic media, and propose an alternative source of spin-orbit coupling to non-paraxial optics vortices. Our treatment puts forth a formulation of nonreciprocal transverse-spin angular-momentum-density shifts for evanescent waves in magneto-optic waveguide media. It shows that magnetization-induced electromagnetic spin-orbit coupling is possible, and that it leads to unequal spin to orbital angular momentum conversion in magneto-optic media evanescent waves in opposite propagation-directions. Generation of free-space helicoidal beams based on this conversion is shown to be spin-helicity- and magnetization-dependent. We show that transverse-spin to orbital angular momentum coupling into magneto-optic waveguide media engenders spin-helicity-dependent unidirectional propagation. This unidirectional effect produces different orbital angular momenta in opposite directions upon excitation-spin-helicity reversals.

The spin-orbit interaction (SOI) of light has been the subject of extensive studies in the last few years^1^,^2^,^3^,^4^,^5^,^6^,^7^,^8^,^9^,^10^,^11^,^12^. Recent experiments have demonstrated strong directional coupling of circularly polarized light (optical spin) in nanophotonic waveguides, where the optical helicity determines the direction of optical flow of the in-coupled light^1^. That is, transversely polarized light acting on nanoparticles on the waveguide cladding can be made to excite beams (optical orbital angular momentum) propagating normal to the incoming wave. The propagation direction of the guided mode being determined by the helicity of the incoming light. Unidirectional surface-plasmon excitation has also been observed in spatially symmetric structures, the surface wave direction switchable with the sense of circularly polarized optical excitation^12^. Other studies have demonstrated optical helicoidal beams, where light in a whispering gallery or ring resonator is made to emit waves possessing orbital angular momentum in free space, as illustrated schematically in Fig. 1^ ^11. Finally, spin-to-orbital angular momentum conversion in tightly focused non-paraxial optical fields in free space has also been demonstrated, where circularly polarized light without a vortex actually exhibits circulating orbital momentum^8^.

This type of spin-orbit interaction in optical wave propagation paves the way to spin-controlled photonics. The use of transverse spin angular momentum and the coupling of transversely propagating circularly polarized beams to waveguide and surface plasmon modes permits selective directional addressing of guided light and quantum states, and enriches the store of spin-dependent tools available to integrated and nano-photonics^1^,^9^,^10^,^11^,^12^.

However, the above studies have not addressed the effects of magneto-optic non-reciprocity on optical spin-orbit coupling, nor ways to induce such coupling by magnetic means. The analysis we present here discusses the generation of optical orbital angular momenta induced through magneto-optic spin-orbit coupling. It analyzes the effect of non-reciprocity on spin-induced transverse optical momenta, as well as magnetization tuning and magnetization reversal effects on unidirectionally spin-induced orbital angular momenta normal to the optical spin. It is known that transverse elliptical polarization of a given helicity occurs in the evanescent tail of optical waveguides, that transversely magnetized magneto-optic waveguides evince a nonreciprocal phase shift, and that the Faraday Effect rotates the polarization of light. Yet the effect of magneto-optic media on the orbital angular momentum shifts in unidirectionally-coupled light upon transverse optical spin reversal, the effect of Faraday rotation upon spin angular momentum conversion and the nonreciprocal transfer to orbital momenta due to electromagnetic spin-orbit coupling, and the magnetic tuning of spin-orbit coupling and its effect on the induced orbital angular momenta have not been addressed. It is these phenomena that are explored in the present report.

Thus, we address the spin-dependent magnetization control of the propagation direction and induced orbital angular momenta. Circularly-polarized beams of a given helicity evanescently-coupled to optical waveguides in the presence of a transverse magnetic field to the optical channel, can be made to switch phase-velocity, alter orbital angular momentum, or cancel unidirectional propagation upon magnetic field tuning, reversals or rotations.

Transverse optical spin is a physically meaningful quantity that can be transferred to material particles^1^,^4^,^5^,^6^,^7^,^8^,^9^. This has potentially appealing consequences for optical-tweezer particle manipulation, or to locate and track nanoparticles with a high degree of temporal and spatial resolution^10^. Thus, developing means of control for the transverse optical spin is of practical interest.

We address the latter question for spin and orbital angular momenta, show that their magnitudes and sense of circulation can be accessed and controlled in a single structure, and propose a specific configuration to this end. Explicit expressions for these physical quantities and for the spin-orbit coupling are presented. Moreover, we develop our treatment for nonreciprocal slab optical waveguides, resulting in a different response upon time reversals.

Consider the behavior of evanescent waves in a magnetic garnet cladding on silicon-on-insulator waveguides, as in Fig. 2. The treatment we present deals with transverse-magnetic (TM) mode propagation. This allows us to obtain explicit expressions for nonreciprocal transverse spin momenta and angular momenta and to propose a means for magnetically controlling these objects, with potential application to integrated optical vortex beam emitters, optical tweezers and quantum computation^9^. The conversion of transverse-spin to orbital angular momenta through spin-orbit coupling relies on TM to transverse-electric (TE) mode conversion. We show that, in this case, mode conversion via Faraday rotation, channels electromagnetic spin-inducing linear momenta into orbital angular momenta that can then be converted into free-space helicoidal beams.

## Results

### Magnetic-Gyrotropy-Dependent Evanescent Waves

The off-diagonal components ±*ig* of the magnetic garnet’s dielectric permittivity tensor, 
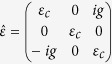
 control the structure’s magneto-optic response. Please refer to the Supplementary Material for the wave equation and dispersion relation for a slab waveguide with magnetic garnet cover layer. The TM mode’s electric-field components in the top cladding are


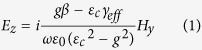



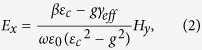


where *H*_*y*_ is the optical magnetic-field, *β* its propagation constant in the z-direction, 
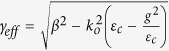
, the decay constant in the (vertical) x-direction, *k*_0_ = 2*π*/*λ, λ* the free-space wavelength, and 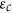 the cover-layer’s dielectric-permittivity constant. The other components, *E*_*y*_ = *H*_*x*_ = *H*_*z*_ = 0.

Notice that these two electric field components are *π*/2 out of phase, hence the polarization is elliptical in the cover layer, with optical spin transverse to the propagation direction. In addition, the polarization evinces opposite helicities for counter-propagating beams, as *E*_*z*_/*E*_*x*_ changes sign upon propagation direction reversal.

This result already contains an important difference with reciprocal non-gyrotropic formulations, where *E*_*z*_/*E*_*x*_ = −*iγ*/*β*, and *γ* the decay constant in the top cladding. Equations 1 and 2 depend on the gyrotropy parameter *g*, both explicitly and implicitly through *β*. and are therefore magnetically tunable, as we shall see below.

We emphasize that the magnitude and sign of the propagation constant *β* change upon propagation direction reversal, and, separately, upon magnetization direction reversal. The difference between forward and backward propagation constants is also gyrotropy dependent. This nonreciprocal quality of magneto-optic waveguides is central to the proper functioning of certain on-chip devices, such as Mach-Zehnder-based optical isolators^13^,^14^.

In a dielectric medium, the momentum density expression accounts for the electronic response to the optical wave. Minkowski’s and Abraham’s formulations describe the canonical and the kinetic electromagnetic momenta, respectively^15^. Here we will focus on Minkowski’s version, 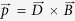, as it is intimately linked to the generation of translations in the host medium, and hence to optical phase shifts, of interest in nonreciprocal phenomena. 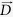 is the displacement vector, and 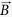 the magnetic flux density.

Dual-symmetric versions of electromagnetic field theory in free space have been considered by various authors^2^,^8^,^9^,^15^. However, the interaction of light and matter at the local level often has an electric character. Dielectric probe particles will generally sense the electric part of the electromagnetic momentum and spin densities^2^,^8^,^9^,^15^. Hence, we treat the standard (electric-biased) formulation of the electromagnetic spin and orbital angular momenta. In the presence of dielectric media, such as iron garnets in the near-infrared range, the expression for Minkowski spin angular momentum becomes


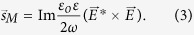


The orbital momentum is


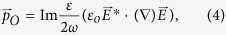


where 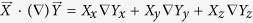, and 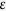 is the relative dielectric permittivity of the medium^3^,^8^. Please refer to the supplementary material for discussion about the origin of these expressions.

In magneto-optic media, the dielectric permittivity 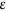 is 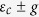, depending on the helicity of the propagating transverse circular polarization. This is usually a small correction to 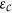, as *g* is two-, or three-, orders of magnitude smaller in iron garnets, in the near infrared range. For elliptical spins, where one helicity component dominates, we account for the admixture level of the minority component in 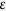 through a weighted average.

### Nonreciprocal Transverse Magneto-Optic Spin-Orbit Coupling

In this section we present a formulation for the transverse-spin and orbital angular momentum densities, and nonreciprocal spin-orbit coupling induced by evanescent fields in magneto-optic media. The magnitude and tuning range of these objects in terms of waveguide geometry and optical gyrotropy are expounded and discussed. We detail the differences in orbital angular momenta between transversely propagating beams induced by circularly-polarized light of opposite helicities. Their unequal response to given optical energy fluxes in opposite propagation directions and to changes in applied magnetic fields are analyzed. And we apply the recently proposed Bliokh-Dressel-Nori electromagnetic spin-orbit correction term to calculate the spin-orbit interaction for evanescent waves in gyrotropic media^8^.

Equations 1 to 4 yield the following expressions for the transverse Minkowski spin angular momentum and the orbital momentum densities in evanescent nonreciprocal electromagnetic waves,


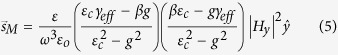



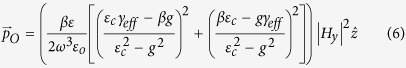


These expressions depend on the magneto-optic gyrotropy parameter *g* and the dielectric permittivity of the waveguide core channel and of its cover layer under transverse magnetization. They yield different values under magnetic field tuning, magnetization and beam propagation direction reversals, and as a function of waveguide core thickness as discussed below. The propagation constant *β* is gyrotropy-, propagation-direction-, and waveguide-core-thickness-dependent, and this behavior strongly impacts the electromagnetic spin and orbital momenta.

Consider now the electromagnetic spin-orbit coupling induced by transversely propagating circularly polarized beams impinging on a gold nanoparticle on the surface of a silicon-on-insulator slab waveguide with Bi:YIG cover layer, as in Fig. 3(a). Alternatively, one may examine the response of a magnetic garnet waveguide on gadolinium-gallium garnet substrate and air cover as in Fig. 3(b). These configurations are similar to the chiral nanophotonic waveguide arrangement considered in ref. 1, except that we are now dealing with a magneto-optic nonreciprocal system. We assume (but do not prove), that the light emitted by the rotating dipole in the gold nanoparticle couples to the elliptically polarized evanescent tail of the same helicity as the rotating dipole, as was shown in refs 1 and 12. The sign of the helicity of the evanescent TM wave locks-in the direction of propagation, resulting in unidirectional spin-orbit coupling.

We now explore the difference in unidirectionally-excited orbital momenta and coupling efficiency for opposite helicities in the magneto-optic system, based on Eq. 5 and Eq. 6. Figure 4(a) plots the shift in coupled orbital momentum per unit spin angular momentum, in a slab waveguide for opposite excitation helicities. This quantity is obtained from the difference in ratio of Eq. 6 to Eq. 5, for opposite propagation directions. The result is plotted as a function of magneto-optic gyrotropy. Plotted in the same figure, Fig. 4(b), we also have the coupling efficiency shift for unidirectional propagation for opposite-helicity circularly polarized excitations. The latter is obtained from the overlap of the circular polarization input to the evanescent tail elliptical polarization, obtained from Eq. 1 and Eq. 2. A derivation of this quantity can be found in the Supplementary Material. Notice that the nonreciprocal orbital momentum shift of the excited unidirectionally-oriented light is significant (0.1%) for typical magneto-optical gyrotropies (~0.001 to 0.01) found in the infrared regime in magnetic garnet materials. Even larger shifts (up to ~1%) obtain in the visible range. Larger shifts are possible for ferromagnetic metallic materials (plasmonic guiding) possessing significantly larger gyrotropies. These latter effects have yet to be explored both theoretically and experimentally.

Consider now the excited nonreciprocal spin angular momentum shifts per energy flow 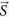,


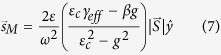


An expression for the energy flow can be found in the Supplementary Material. Figure 2 plots the nonreciprocal transverse spin-angular-momentum-density shift per unit energy flux, as a function of silicon slab thickness in an SOI slab waveguide with Ce_1_Y_2_Fe_5_O_12_ garnet top cladding. Calculations are performed for the same electromagnetic energy flux in opposite propagation directions, at a wavelength of 1550 nm, *g* = −0.0086. The nonreciprocal shift is normalized to the average spin angular momentum. The energy shift evinces a relatively stable value, close to 0.7% above 0.3 μm thickness. Its thickness dependence is a function of the ellipticity of the transverse polarization in the x-z plane. Above 0.3 μm, the ellipticity ranges from 31.4° to 36.9°, where 45° corresponds to circular polarization. In other words, the ellipticity stays fairly constants, with a moderately small admixture of the minority circularly polarized component, ranging from 25% to 14%. Below 0.3 μm, the minority component admixture increases precipitously, reaching 87% at 0.13 μm. Magnetization reversals produce the same effect, for the corresponding transverse spin-angular-momentum-density shift. Refer to the Supplementary Material for full expression.

### Magneto-Optic Gyrotropy Control of Spin-Orbit Effects

The magneto-optic gyrotropy of an iron garnet can be controlled through an applied magnetic field. These ferrimagnetic materials evince a hysteretic response, such as the one displayed in Fig. 5 (inset) for 532 nm wavelength in a sputter-deposited film. The target composition is Bi_1.5_Y_1.5_Fe_5.0_O_12_. Shown here are actual experimental data extracted from Faraday rotation measurements. Below saturation, the magneto-optic response exhibits an effective gyrotropy value that can be tuned through the application of a magnetic field. These measurements correspond to a 0.5 μm-thick film on a (100)-oriented gadolinium gallium garnet (GGG) substrate. The optical beam is incident normal to the surface, and the hysteresis loop probes the degree of magnetization normal to the surface as a function of applied magnetic field. These data show that the electromagnetic spin angular momentum can be tuned below saturation and between opposite magnetization directions.

Figure 5 also reveals an interesting feature about the magneto-optic gyrotropy. The normalized nonreciprocal transverse spin-angular-momentum-density shift per unit energy flux linearly tracks the gyrotropy, and is of the same order of magnitude as *g*, although thickness-dependent. Yet, as pointed out before, this thickness dependence reflects the admixture of the minor helicity component in the spin ellipticity. At 0.4 μm, for example, 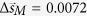 when *g* = −0.0086. However, the major polarization helicity component contribution to 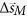 is 84.4% at this thickness, translating into 0.00853 at 100%. At 0.25 μm, 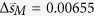, and the major polarization helicity component contribution is 76.2%, translating into 0.0086 at 100%. *We, thus, re-interpret the magneto-optical gyrotropy as the normalized spin-angular-momentum density shift per unit energy flux.*

**Figure 1:**
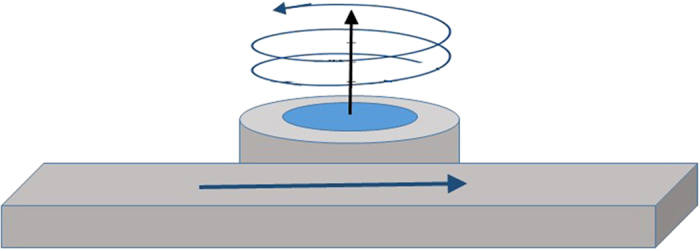
Schematic depiction of a micro-ring optical resonator coupled to a feeder waveguide used to emit helicoidal waves possessing orbital angular momentum into free space.

**Figure 2:**
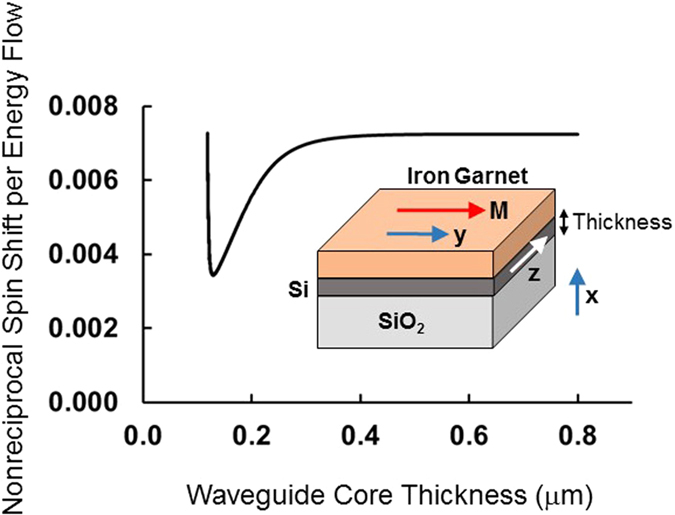
Normalized nonreciprocal Minkowski transverse spin-angular-momentum-density shift per unit energy flux as a function of silicon slab thickness for *g* = −0.0086, corresponding to Ce_1_Y_2_Fe_5_O_12_ garnet top cladding on SOI at *λ* = 1.55 *μm* wavelength. The inset shows the slab waveguide structure. **M** stands for the magnetization in the garnet.

**Figure 3:**
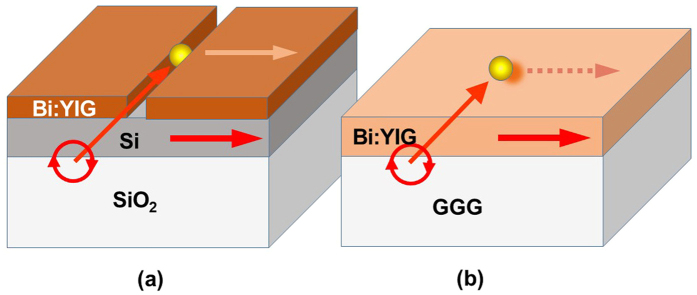
Schematic depiction of electromagnetic spin-orbit coupling configuration consisting of a transversely-propagating circularly polarized beam impinging on a gold nanoparticle on (**a**) the surface of a silicon-on-insulator slab waveguide with bismuth-substituted yttrium iron garnet (Bi:YIG) cover layer, or (**b**) a Bi:YIG slab waveguide, to produce unidirectional propagation of waveguide modes normal to the incoming beam.

**Figure 4:**
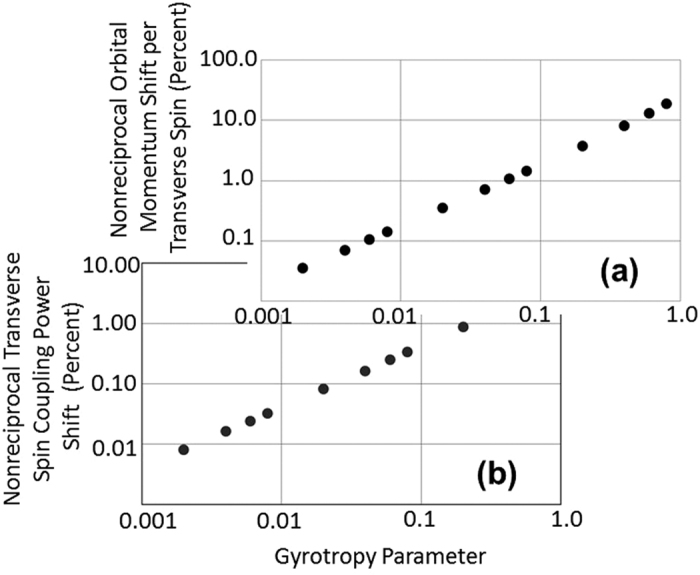
Difference in (**a**) unidirectionally-excited orbital momenta and (**b**) coupling efficiencies for opposite helicities in the magneto-optic system. 4(**a**) plots the shift in coupled orbital momentum per unit spin angular momentum, in a slab waveguide for opposite excitation helicities.

**Figure 5:**
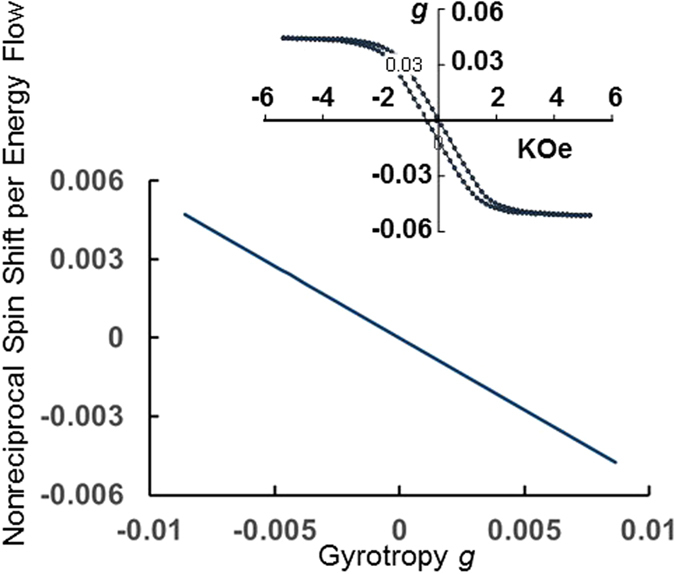
Normalized nonreciprocal Minkowski transverse spin-angular-momentum-density shift per unit energy flux as a function of magneto-optical gyrotropy. Data correspond to 0.25 μm silicon-slab thickness with Ce_1_Y_2_Fe_5_O_12_ garnet top cladding, *λ* = 1.55 *μm* wavelength. The inset shows the gyrotropy versus magnetic field hysteresis loop of a magnetic garnet film at *λ* = 532 *nm*, sputter-deposited using a Bi_1.5_Y_1.5_Fe_5.0_O_12_ target.

Figure 1 illustrates schematically the induction of free-space vortex beams. The difference in free-space vortex beams orbital angular momenta are therefore apparent from Fig. 4, a consequence of the difference induced by coupling light with positive or negative spin helicities into the waveguide. The fact that the magneto-optic gyrotropy can be tuned, as discussed above, means that the magnitude orbital momenta and the phase of the coupled light (and hence the resonance of the coupled light in the resonator), can be tuned to resonate for either positive or negative helicity excitation beams, while at the same time suppressing the excitation of one or the other free-space helicoidal beams. These are novel proposed effects that translate into magnetic control of free-space helicoidal beams for opposite chiralities.

### Magnetization-Induced Electromagnetic Spin-Orbit Coupling

Bliokh and co-authors have studied the electromagnetic spin-orbit coupling in non-paraxial optical beams^8^. They find that there is a spin dependent term in the orbital angular momentum expression that leads to spin-to-orbit angular momentum conversion. This phenomenon occurs under tight focusing or the scattering of light^8^. Here we consider an alternative source of electromagnetic spin-orbit coupling, magnetization-induced coupling in evanescent waves.

The time-averaged spin-, 
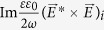
, and orbital-, 
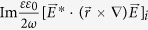
, angular momenta conservation laws put forth in ref. 8 each contain a term, responsible for spin-orbit coupling, in the form


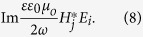


**Figure 6:**
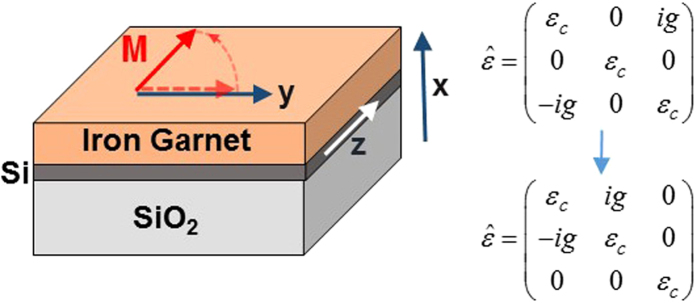
Rotated magnetization M generates TM to TE waveguide mode coupling and electromagnetic spin-orbit coupling. The figure also shows the electric permittivity tensor before and after rotation.

We have modified the original expressions to include a dielectric permittivity factor 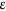 to account for the material response of the medium. The indices *i* = *x, y, z*. Expression 8 appears with opposite signs in the spin and orbital conservation laws, signaling a transfer of angular momentum from spin to orbital motion. As it stands, so far in our treatment, this term equals zero, since the spin points in the y-direction and the electric-field components of the TM wave point in the *x*-, and *z*-directions. A way to overcome this null coupling, and enable the angular momentum transfer, is to rotate the applied magnetic field about the *x*-axis away from the *y*-direction, as in Fig. 6. This action induces a Faraday rotation about the *z*-axis, generating a spin-orbit coupling term in the angular momentum conservation laws. An in-plane rotation of the magnetization **M** to the z-axis will induce TM to TE mode conversion and electromagnetic spin-orbit interaction in the magneto-optic medium^16^,^17^. Hence, non-zero electromagnetic field components 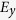 and 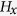, and spin-orbit coupling, are induced in the propagating wave. The spatial, non-intrinsic, component, characteristic of orbital motion, emerges in the form of a *z*-dependence in the angular momentum, embodied in the partial or total evanescence of the major circularly-polarized transverse-spin component as the wave propagates along the guide.

In what sense is there an angular momentum transfer from spin to orbital, in this case? As the polarization rotates in the x-y plane due to the Faraday Effect, there will be a spatially-dependent reduction in the circulating electric field spin-component of the electromagnetic wave along the propagation-direction. This can be seen as a negative increase in circular polarization with z, i.e., an orbital angular momentum in the opposite direction to the electromagnetic spin.

More specifically, the TE mode, with an electric-field component only in the *y*-direction, carries no transverse spin angular momentum, as per Eq. 3. Where does this angular momentum go? It goes into orbital angular momentum, according to Eq. 8. This electromagnetic orbital angular momentum (OAM) in the TE mode may be converted into free-space OAM via a helicoidal beam emitter as proposed in ref. 11. These authors demonstrate an integrated compact vortex beam emitter through the use of a circular micro-ring or micro-disk optical resonator, as in Fig. 1, furnished with an embedded angular grating. The grating partially converts the whispering gallery mode in the micro-ring into free-space radiation. The device is configured to emit vortex beams from quasi-TE light fed into the micro-ring via a straight waveguide bus coupled to it. At issue here is not the Faraday rotation per se, but the conversion of transverse spin angular momentum into orbital angular momentum.

Finally, we derive an explicit expression for the spin-orbit coupling term. We assume that Faraday rotation induces the *E*_*y*_, *H*_*z*_ terms via TM to TE mode conversion, where 
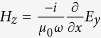
, and 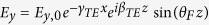. 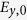 is the electric field amplitude corresponding to full TM to TE conversion, 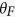 is the specific Faraday rotation angle, 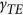 and 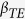 are the cover-layer decay constant and the propagation constant for the TE mode, respectively. For simplicity, we assume no linear birefringence in the waveguide, so 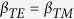. The spin to orbital angular-momentum coupling term is then


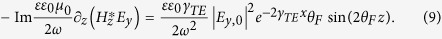


Hence full angular momentum conversion from transversely-coupled forward (or backward) propagating positive (negative) helicity light in the z-direction upon 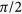 Faraday rotation generates a spin-to orbital momentum transfer 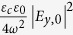 in the cover layer.

## Discussion

There are two key results put forth in this article. A formulation of the interaction between nonreciprocal transverse electromagnetic spin and orbital angular momenta in evanescent waves in magneto-optic media. And a treatment of magnetically induced spin-orbit coupling in electromagnetic waves. Our analysis examines the effect of magneto-optic nonreciprocity on the orbital angular momenta of unidirectional light in nanophotonic waveguide interfaces generated by electromagnetic spin-orbit interaction. We explore the role of optical chirality on the induced orbital momenta on transversely propagating light and quantify its response to opposite-spin optical excitations. The dependence of induced orbital momenta on magneto-optical gyrotropy is detailed.

Additionally, the results presented here provide a means for testing the Bliokh-Dressel-Nori electromagnetic spin-orbit coupling formulation in magneto-optic media^8^. And, simultaneously, offer a way for magnetically inducing and controlling the electromagnetic spin-orbit interaction. The approach may be used to generate OAM helicoidal beams through spin to orbital angular momentum conversion, and to magnetically tune the production of free space orbital angular momenta via ring resonators. The latter relies on experimental results already demonstrated by X. Cai and co-workers in ref. 11.

Our treatment of the nonreciprocal transverse electromagnetic spin is developed for slab waveguides with magnetic garnet cladding layers. This approach allows for an explicit analytical solution of the spin and orbital momenta and angular momenta that can be experimentally tested via prism-coupling in slab waveguides. As such, it allows for testing the controversial reality of electromagnetic spin momenta, in other words, the reality of the electromagnetic linear momenta that induce transverse spin-angular momenta.

The article shows that transverse spin angular momentum in evanescent waves can be magnetically tuned, with possible applicability to nanoparticle optical manipulation, and that it evinces a precisely quantifiable nonreciprocal response in magneto-optic media. Moreover, we demonstrate that the shift in spin angular momentum per unit energy flow upon time or magnetization reversal corresponds to the magneto-optical gyrotropy. This finding provides a dynamic interpretation of the magneto-optic gyrotropy parameter, and gives a fresh perspective on the source of the nonreciprocal phase shift effect, often used in the design of integrated optical isolators.

The thickness dependence of the nonreciprocal transverse-spin-shift upon time or magnetization reversals is found to reflect the admixture of minority circular polarization component in the elliptical spin configuration in evanescent waves. This admixture is very pronounced for thin slabs near cutoff, but wanes and saturates for thicker samples, where the majority circular polarization dominates.

Our treatment of electromagnetic spin-orbit coupling also provides a means for magnetically inducing and modulating OAM and free-space helicoidal beams. These can be produced on-chip, in magneto-optic waveguides with magneto-optic claddings, thus allowing for compact packaging of vortex-bean sources. Nonreciprocal TE to TM mode conversion in semiconductor waveguides with magneto-optic upper claddings has already been demonstrated^16^,^17^.

Numerous applications of optical angular momentum and vortex beams have been discussed in the literature. These include their use in optical tweezers^18^,^19^, optical microscopy^20^, and quantum and wireless communications^21^,^22^.

## Method

The theoretical tools to develop the formulation and angular momenta expressions presented here are simple differential equations and algebraic manipulation. C++ computer programming is used to evaluate waveguide propagation constants based on modal dispersion, and to compute spin and orbital angular momenta in evanescent waves in the garnet cladding. These calculations are used to analyze the nonreciprocal response of the spin and orbital angular momenta as a function of slab waveguide thickness. Radio-frequency magnetron sputter-deposition is used to fabricate bismuth-substituted yttrium iron garnet films on gadolinium gallium garnet substrates, and a rotating polarizer setup is used to measure their Faraday rotation hysteresis.

## Additional Information

**How to cite this article**: Levy, M. and Karki, D. Nonreciprocal Transverse Photonic Spin and Magnetization-Induced Electromagnetic Spin-Orbit Coupling. *Sci. Rep.* **7**, 39972; doi: 10.1038/srep39972 (2017).

**Publisher's note:** Springer Nature remains neutral with regard to jurisdictional claims in published maps and institutional affiliations.

## Supplementary Material

Supplementary Material
