# Supplementary material for: Nonreciprocal Transverse Photonic Spin and Magnetization-Induced Electromagnetic Spin-Orbit Coupling
Source: Sci Rep. 2017 Jan 6;7:39972. doi: 10.1038/srep39972 (PMC5216357; doi:10.1038/srep39972)
Supplement: Supplementary Material [file srep39972-s1.pdf]

Supplementary Material for Nonreciprocal Transverse Photonic Spin and Magnetization-Induced Electromagnetic Spin-Orbit Coupling

Miguel Levy* and Dolendra Karki

Physics Department, Michigan Technological University

Henes Center for Quantum Phenomena, Michigan Technological University

*mlevy@mtu.edu

In 1939, F. J. Belinfante introduced a spin momentum density expression for vector fields to explain the spin of quantum particles and symmetrize the energy-momentum tensor [1]. For monochromatic electromagnetic waves in free-space, the corresponding spin linear-momentum density reads

$$\vec{p}_B = \frac{1}{2}\vec{\nabla}\times\vec{s}_B, \tag{S1}$$

with a time-averaged spin angular-momentum density

$$\vec{s}_B = \mathrm{Im}\frac{1}{2\omega}(\varepsilon_o\vec{E}^*\times\vec{E}). \tag{S2}$$

ω is the optical frequency and ε_0 the permittivity of free-space [2].

The optical spin-angular-momentum density, Eq. S2, is derivable from the expression for the total electromagnetic angular momentum in terms of Poynting's vector, [3]

$$\int\vec{r}\times[\varepsilon_0\mu_0\vec{E}\times\vec{H}]d^3r = \int\vec{r}\times[\varepsilon_0\vec{E}\times(\nabla\times\vec{A})]d^3r\ . \tag{S3}$$

$\varepsilon_o \mu_0 \vec{E} \times \vec{H}$ is the electromagnetic momentum density, and $\vec{A}$ the vector potential. Upon integration by parts, the integrand in Eq. 3 acquires an "intrinsic" term, $\varepsilon_0 \vec{E} \times \vec{A}$, independent of radius vector $\vec{r}$ except, implicitly, through the field components. This term is usually associated with the electromagnetic spin density [3]. The remaining term

$$\sum_{i=x,y,z} E_i \left(\vec{r} \times \vec{\nabla} \right) A_i \tag{S4}$$

corresponds to the orbital angular momentum density.

This spin angular momentum, in its transverse electromagnetic form, has merited much attention in recent years, as it can be studied in evanescent waves [4-8]. There are fundamental and practical reasons for this.

Until recently, the quantum field theory of the electromagnetic field has lacked a description of separate local conservation laws for the spin and orbital angular momentum-generating currents [8]. Whether such spin-generating momenta, as opposed to the actual spin angular momenta they induce, are indeed observable or merely 'virtual' is of fundamental interest.

Moreover, if the electromagnetic spin and orbital momenta are separable, the question arises as to whether there are any photonic spin-orbit interaction effects. In other words, is it possible to convert electromagnetic spin into orbital angular momentum and vice-versa? Bliokh, Dressel and Nori give a positive answer for non-paraxial fields [8]. Using the conservation laws proposed by these authors, we show here that it is also possible to magnetically induce electromagnetic spin-orbit coupling in magneto-optic media. And we demonstrate, specifically, that Faraday rotation can be relied upon to convert the transverse spin of evanescent waves in guided light into orbital angular momentum of free-space beams, thus confirming the validity of the Bliokh-Dressel-Nori

formulation, and providing a mechanism for free-space optical angular momentum generation and control. We show that the transfer of spin-generating momenta into orbital momenta plays a central role in electromagnetic spin-orbit coupling.

The electromagnetic field-expressions for transverse magnetization (y-direction) and monochromatic TM mode propagation in the z-direction in a slab waveguide are,

$$\vec{E} = \left(E_x, 0, E_z\right)\mathrm{e}^{i(\beta z-\omega t)} \tag{S5}$$

$$\vec{H} = \left(0, H_y, 0\right)e^{i(\beta z-\omega t)} \tag{S6}$$

In magneto-optic media, the off-diagonal components $\pm ig$ of the dielectric permittivity tensor $\hat{\varepsilon}$, parameterize the magneto-optic gyrotropy. Maxwell-Ampere's and Faraday's laws in ferrimagnetic media are

$$\vec{\nabla}\times\vec{H} = \varepsilon_o\hat{\varepsilon}\frac{\partial\vec{E}}{\partial t} = \varepsilon_o\begin{pmatrix}\varepsilon_c & 0 & ig\\ 0 & \varepsilon_c & 0\\ -ig & 0 & \varepsilon_c\end{pmatrix}\frac{\partial\vec{E}}{\partial t} = -\varepsilon_o\begin{pmatrix}\varepsilon_c & 0 & ig\\ 0 & \varepsilon_c & 0\\ -ig & 0 & \varepsilon_c\end{pmatrix}i\omega\vec{E} \tag{S7}$$

$$\vec{\nabla}\times\vec{E} = -\mu_o\frac{\partial\vec{H}}{\partial t} = \mu_o i\omega\vec{H} \tag{S8}$$

We examine transverse-magnetic (TM) propagation in the slab. Vertical and transverse-horizontal directions are x, and y, respectively, β is the propagation constant, and the wave equation in the iron garnet is given by

$$\frac{\partial^2}{\partial x^2}H_y + \left[k_o^2\left(\varepsilon_c - \frac{g^2}{\varepsilon_c}\right) - \beta^2\right]H_y = 0 \text{ , with } k_0 = 2\pi/\lambda \text{, for wavelength } \lambda \text{ [9].} \tag{S9}$$

we get:

$$H_y = H_c e^{-\gamma_{eff}x}, x > 0 \qquad \text{(Top cladding)} \tag{S10}$$

$$H_y = H_f \cos(k_x x + \phi_c), -d < \mathrm{x} < 0 \qquad \text{(Core)} \qquad \text{(S11)}$$

$$H_y = H_s \exp(\gamma_s (x+d)), x < -d \ , \qquad \text{(Substrate)} \qquad \text{(S12)}$$

where

$$\gamma_{eff} = \sqrt{\beta^2 - k_o^2 \varepsilon_{eff}}$$

$$k_x = \sqrt{k_o^2 \varepsilon_f - \beta^2} \ , \qquad \text{(S13)}$$

$$\gamma_s = \sqrt{\beta^2 - k_o^2 \varepsilon_s} \qquad \text{(S14)}$$

ε_f , and ε_s are the silicon-slab and substrate dielectric-permittivity constants, respectively, and d is the slab thickness.

Defining

$$\varepsilon_{eff} = \left(\varepsilon_c - \frac{g^2}{\varepsilon_c} \right) \qquad \text{(S15)}$$

as an effective permittivity in the cover layer, and

$$\gamma_{eff} = \sqrt{\beta^2 - k_o^2 \varepsilon_{eff}} \ , \qquad \text{(S16)}$$

as the corresponding decay constant in the x-direction, one finds,

$$E_z = i \frac{g\beta - \varepsilon_c \gamma_{eff}}{\omega \varepsilon_0 (\varepsilon_c{}^2 - g^2)} H_y \qquad \text{(S17)}$$

$$E_x = \frac{\beta \varepsilon_c - g \gamma_{eff}}{\omega \varepsilon_0 (\varepsilon_c{}^2 - g^2)} H_y \, , \qquad \text{(S18)}$$

We treat the standard (electric-biased) formulation of the electromagnetic spin and orbital angular momenta. In the presence of dielectric media, such as iron garnets in the near-infrared range, the expression for spin angular momentum becomes

$$\vec{s}_M = \mathrm{Im}\frac{\varepsilon_o \varepsilon}{2\omega}(\vec{E}^* \times \vec{E}) . \qquad \text{(S19)}$$

The orbital momentum is

$$\vec{p}_O = \mathrm{Im}\frac{\varepsilon}{2\omega}(\varepsilon_o \vec{E}^* \cdot (\nabla)\vec{E}) \text{ , where} \qquad \text{(S20)}$$

$\vec{X} \cdot (\nabla)\vec{Y} = X_x \nabla Y_x + X_y \nabla Y_y + X_z \nabla Y_z$, and ε is the relative dielectric permittivity of the medium [3, 8]. This expression is derivable from Eq. S4.

The transverse Minkowski spin angular momentum, spin momentum and the orbital momentum densities in evanescent nonreciprocal electromagnetic waves, derived from Eq. S17, Eq. S18, Eq. S19, and Eq. S20, are

$$\vec{s}_M = \frac{\varepsilon}{\omega^3 \varepsilon_o}\left(\frac{\varepsilon_c \gamma_{eff} - \beta g}{\varepsilon_c^2 - g^2}\right)\left(\frac{\beta \varepsilon_c - g\gamma_{eff}}{\varepsilon_c^2 - g^2}\right)\left|H_y\right|^2 \hat{y} \qquad \text{(S21)}$$

$$\vec{p}_M = -\frac{\varepsilon \gamma_{eff}}{\omega^3 \varepsilon_o}\left(\frac{\varepsilon_c \gamma_{eff} - \beta g}{\varepsilon_c^2 - g^2}\right)\left(\frac{\beta \varepsilon_c - g\gamma_{eff}}{\varepsilon_c^2 - g^2}\right)\left|H_y\right|^2 \hat{z} \qquad \text{(S22)}$$

$$\vec{p}_O = \left(\frac{\beta \varepsilon}{2\omega^3 \varepsilon_o}\left[\left(\frac{\varepsilon_c \gamma_{eff} - \beta g}{\varepsilon_c^2 - g^2}\right)^2 + \left(\frac{\beta \varepsilon_c - g\gamma_{eff}}{\varepsilon_c^2 - g^2}\right)^2\right]\right)\left|H_y\right|^2 \hat{z} \qquad \text{(S23)}$$

And the ratio

$$\left|\frac{\vec{p}_O}{\vec{s}_M}\right| = \frac{\beta}{2}\left(\frac{\varepsilon_c\gamma_{eff} - \beta g}{\beta\varepsilon_c - g\gamma_{eff}} + \frac{\beta\varepsilon_c - g\gamma_{eff}}{\varepsilon_c\gamma_{eff} - \beta g}\right) \tag{S24}$$

These expressions depend on the magneto-optic gyrotropy parameter g and the dielectric permittivity of the waveguide core channel and of its cover layer under transverse magnetization. They yield different values under magnetic field tuning, magnetization and beam propagation direction reversals, and as a function of waveguide core thickness as discussed below. The propagation constant β is gyrotropy-, propagation-direction-, and waveguide-core-thickness-dependent, and this behavior strongly impacts the electromagnetic spin and orbital momenta. Re-expressing the transverse Minkowski spin angular momentum and spin momentum densities in terms of the energy flow $\vec{S}$,

$$\vec{s}_M = \frac{2\varepsilon}{\omega^2}\left(\frac{\varepsilon_c\gamma_{eff} - \beta g}{\varepsilon_c^2 - g^2}\right)\left|\vec{S}\right|\hat{y} \tag{S25}$$

$$\vec{p}_M = -\frac{2\varepsilon\gamma_{eff}}{\omega^2}\left(\frac{\varepsilon_c\gamma_{eff} - \beta g}{\varepsilon_c^2 - g^2}\right)\vec{S} \tag{S26}$$

The time-averaged electromagnetic energy flux (Poynting's vector) in the iron garnet layer is

$$\vec{S} = \tfrac{1}{2}\mathrm{Re}(\vec{E}^* \times \vec{H}) = \frac{1}{2}\frac{\beta\varepsilon_c - g\gamma_{eff}}{\omega\varepsilon_0(\varepsilon_c{}^2 - g^2)}\left|H_y\right|^2\hat{z}\,. \tag{S27}$$

The nonreciprocal shift normalized to the average spin angular momentum is expressed as follows,

$$\Delta\vec{s}_M = \frac{2\left[\varepsilon_f\left(\varepsilon_c\gamma_{eff} - \beta g\right)_f - \varepsilon_b\left(\varepsilon_c\gamma_{eff} - \beta g\right)_b\right]}{\varepsilon_f\left(\varepsilon_c\gamma_{eff} - \beta g\right)_f + \varepsilon_b\left(\varepsilon_c\gamma_{eff} - \beta g\right)_b}\,. \tag{S28}$$

Subscripts f and b stand for forward, and backward propagation, respectively.

Figure 1 plots the Minkowski transverse spin-angular-momentum-density shift, as a function of silicon slab thickness. Specifically, it shows the normalized shift in the Eq. 16 pre-factor,

$$\Delta s_M = \frac{\left[\varepsilon\left(\frac{\varepsilon_c\gamma_{eff}-\beta g}{\varepsilon_c^2-g^2}\right)\left(\frac{\beta\varepsilon_c-g\gamma_{eff}}{\varepsilon_c^2-g^2}\right)\right]_g-\left[\varepsilon\left(\frac{\varepsilon_c\gamma_{eff}-\beta g}{\varepsilon_c^2-g^2}\right)\left(\frac{\beta\varepsilon_c-g\gamma_{eff}}{\varepsilon_c^2-g^2}\right)\right]_{-g}}{\frac{1}{2}\left\{\left[\varepsilon\left(\frac{\varepsilon_c\gamma_{eff}-\beta g}{\varepsilon_c^2-g^2}\right)\left(\frac{\beta\varepsilon_c-g\gamma_{eff}}{\varepsilon_c^2-g^2}\right)\right]_g+\left[\varepsilon\left(\frac{\varepsilon_c\gamma_{eff}-\beta g}{\varepsilon_c^2-g^2}\right)\left(\frac{\beta\varepsilon_c-g\gamma_{eff}}{\varepsilon_c^2-g^2}\right)\right]_{-g}\right\}} \quad \text{(S29)}$$

We observe a moderate, and relatively stable, admixture of minority circularly-polarized component above 0.3μm thickness.

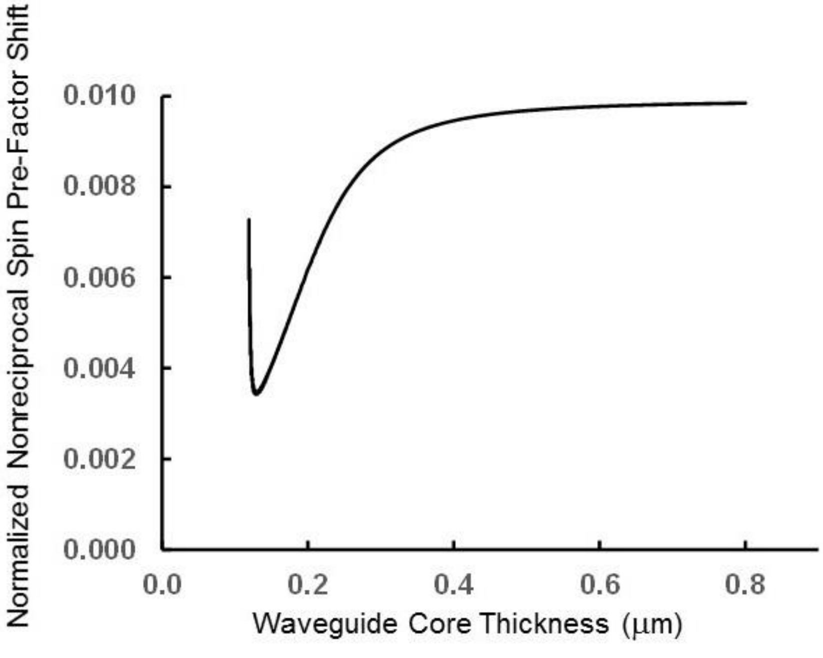

Fig. 1. Normalized nonreciprocal Minkowski transverse spin-angular-momentum-density pre-factor shift as a function of silicon slab thickness for $g=-0.0086$, corresponding to $Ce_1Y_2Fe_5O_{12}$ garnet top cladding on SOI at $\lambda=1.55\mu m$ wavelength.

Finally, in order to estimate the coupling of circularly polarized light in an elliptical mode, we express the incoming circular polarization as the superposition of elliptical normal modes of opposite helicities, and take the fraction that couples into the same helicity elliptical mode to be the amplitude fraction of the circularly polarized incoming beam that gets coupled in, as follows:

$$E_+ \frac{1}{\sqrt{2}}(\hat{x}+i\hat{z}) = E_{+e}\frac{(\hat{x}+ie\hat{z})}{\sqrt{1+e^2}} + E_{-e}\frac{(e\hat{x}-i\hat{z})}{\sqrt{1+e^2}} \tag{S30}$$

This yields:

$$\left(\frac{E_{+e}}{E_+}\right)^2 = \left(\frac{1}{2}\right)\frac{(e+1)}{1+e^2} \tag{S31}$$

References

1. Belinfante, F. J. On the current and the density of electric charge, the energy, the linear momentum and the angular momentum of arbitrary fields. Physica 7, 449-474 (1940).

2. Bliokh, K. Y., Bekshaev, A. Y. & Nori, F. Extraordinary momentum and spin in evanescent waves. Nature Communications **5**, 4300 (2014).

3. Barnett, S. M. Rotation of electromagnetic fields and the nature of optical angular momentum. J. of Modern Optics **57**, 1339-1343 (2010).

4. Neuberger, M. *et. al.* Experimental demonstration of the geometric so in Hall effect of light in highly focused vector beams. Conference on Lasers and Electro-Optics," QW1E.4 (OSA 2012).

5. Banzer, P. *et. al.* The photonic wheel demonstration of a state of light with purely transverse momentum," J. Eur. Opt. Soc. Rap. Publ. **8**, 13032 (2013).

6. Bliokh, K. Y. & Nori, F. Transverse spin of a surface polariton. Phys. Rev. A **85**, 061801 (2012).

7. Kim, K. –Y., Lee, I. –M., Kim, J., Jung, J. & Lee, B. Time reversal and the spin angular momentum of transverse electric and transverse-magnetic surface modes. Phys. Rev. A **86**, 063805 (2012).

8. Bliokh, K. Y., Dressel J., & Nori, F. Conservation of the spin and orbital angular momenta in electromagnetism. New Journal of Physics **16**, 093037 (2014).

9. Fujita, J., Levy, M., Osgood, Jr., R. M., Wilkens, L. & Dötsch, H. Waveguide optical isolator based on Mach-Zehnder interferometer. Appl. Phys. Lett. **76**, 2158 (2000).
